# Fast, Low-Cost and Non-Destructive Physico-Chemical Analysis of Virgin Olive Oils Using Near-Infrared Reflectance Spectroscopy

**DOI:** 10.3390/s17112642

**Published:** 2017-11-16

**Authors:** Ana Garrido-Varo, María-Teresa Sánchez, María-José De la Haba, Irina Torres, Dolores Pérez-Marín

**Affiliations:** 1 Department of Animal Production, Faculty of Agricultural and Forestry Engineering, University of Cordoba, Campus Rabanales, E-14071 Cordoba, Spain; dcperez@uco.es; 2 Department of Bromatology and Food Technology, Faculty of Agricultural and Forestry Engineering, University of Cordoba, Campus Rabanales, E-14071 Cordoba, Spain; bt1hacem@uco.es (M.-J.D.l.H.); g72toroi@uco.es (I.T.)

**Keywords:** near-infrared spectroscopy, olive oil, physico-chemical quality, MPLS regression, analysis mode

## Abstract

Near-Infrared (NIR) Spectroscopy was used for the non-destructive assessment of physico-chemical quality parameters in olive oil. At the same time, the influence of the sample presentation mode (spinning *versus* static cup) was evaluated using two spectrophotometers with similar optical characteristics. A total of 478 olive oil samples were used to develop calibration models, testing various spectral signal pre-treatments. The models obtained by applying MPLS regression to spectroscopic data yielded promising results for olive oil quality measurements, particularly for acidity, the peroxide index and alkyl and ethyl ester content. The results obtained indicate that this non-invasive technology can be used successfully by the olive oil sector to categorize olive oils, to detect potential fraud and to provide consumers with more reliable information. Although both sample presentation modes yielded comparable results, equations constructed with samples scanned using the spinning mode provided greater predictive capacity.

## 1. Introduction

Virgin olive oil is an essential component of the traditional Mediterranean diet. Its quality is known to be influenced by a number of factors, including plant variety, environmental conditions, agronomic and harvesting practices and technological processes [1,2].

The International Olive Council (IOC) [3] and the European Union (EU) [4] have established standards that can be applied to all the olive oils subject to international trade. The Commission Delegated Regulation (EU) 2015/1830 recognizes four categories of olive oil—Extra Virgin (EVOO), Virgin (VOO), Lampante (LOO) and Refined (ROO). Extra virgin is the most expensive and health-promoting of all the categories of olive oil, while Lampante olive oil is reserved for refining and technical purposes, its direct use for human consumption being prohibited. The EU directive governing the characterization of olive oil specifies 26 physico-chemical and two sensory characteristics of these oils, prominent among the physico-chemical parameters being acidity, the peroxide index, extinction coefficients at 232 and 270 nm (K_232_ and K_270_), indicators of changes in the fruit, inappropriate fruit handling and deficient fruit or oil storage conditions as well as oxidation of the oil and its state of preservation [5]. In addition, alkyl and ethyl esters are compounds deriving from non-desired fermentation of the olives and therefore represent a deterioration in quality. Determining the level of alkyl esters (fundamentally composed of ethyl esters but also methyl esters) is of great utility for distinguishing between olive oils and olive pomace oils and for detecting the fraudulent mixture of extra virgin olive oils with low-quality oils, whether lampante oils or oils that have been deodorized at low temperature [6].

Despite the huge amount of effort invested in research into physico-chemical and sensory methods for determining the quality, purity and authenticity of virgin olive oils and the international standards that are used in official inspections by the various producer and importer countries, the adulteration of extra virgin olive oil with low-grade oils remains a major international problem, with considerable repercussion in the media. In the present authors’ view, the main factor accounting for the recurring episodes of fraud is the inspection bodies’ lack of a suitable and affordable methodology that would significantly increase the number of samples inspected per year, providing substantially more evidence than is currently available. There is a need for exhaustive analytical inspections to be carried out on a large volume of olive oils traded worldwide. 

Furthermore, although all the analytical parameters provide valuable information on the oils in question, many of them continue to be inaccessible to the majority of producers and retailers. It is for this reason that the obligatory labelling of oils provides only limited information of interest to consumers [7]. 

In recent years, a wide set of fast analytical techniques such as UV-Vis spectroscopy, fluorescence, vibrational spectroscopies (NIR, MIR and Raman fluorescence) together with chemometric algorithms has been used to differ samples by cultivar/origin, to guarantee the authenticity of olive oil and/or detect adulteration with other oils [8]. In this sense, Near-Infrared (NIR) spectroscopy has proved to be ideally suited to the analysis of thousands of samples per day. This technology has the capacity to analyze a sample for multiple constituents in a fraction of a second or 2–3 min (depending on the instrument); it also involves a low per-sample cost, little or no sample preparation and the possibility of analyzing samples at-line, on-line, in-line and in the field (i.e. olive trees, soil, leaves, etc.) without any utilization, handling or disposal of chemicals and chemical waste. 

NIR applications in fats and oils have been thoroughly reviewed [7,8]. The authors make it clear that most of the work carried out in the specific case of olive oil (OO) in the period under review is concerned with viability trials—using part or all of the NIR region (1100–2500 nm)—geared towards the detection of mixtures and adulterations and the detection of geographical origins. Garrido-Varo et al. [9] published the first work on the application of NIR spectroscopy for quantifying the physico-chemical and sensory parameters of olive oil, demonstrating the feasibility of Vis/NIR spectroscopy to accurately predict such properties as acidity, specific extinction coefficients (K_225_, K_232_, K_270_), polyphenols, oxidative stability and, with less precision, other parameters such as the peroxide index and moisture. They additionally concluded that Vis/NIR technology could produce excellent predictions (*r*^2^_cv_ = 0.88; SECV = 0.50) for OO samples graded as 2.20–7.12, categorized by their organoleptic characteristics on a 9-point scale (9 for exceptional EVOO and 1 for LOO) [10]. More recently, the application of NIR spectroscopy to olive oil has also been reviewed by Casale and Simonetti [11], who confirmed the suitability of NIR spectroscopy for predicting other characteristics, such as its fruity, bitter and pungent attributes, the linoleic, linolenic, stearic and oleic acid content and the content of α-tocopherol. These authors conclude that despite the existence of positive scientific findings, the use of NIR spectroscopy in the olive oil industry is still in its infancy. 

It is generally accepted that before any NIR applications can be adopted by the industry and by official inspection laboratories, many variables affecting the quality of the spectra stand in need of scientific evaluation. In the case of liquids, the way in which samples are presented to the instrument has been identified as one of the critical aspects needing to be optimized. NIR analysis of liquids can be done by transmission (using a quartz cuvette or a disposable glass vial) or transflectance (using a gold/aluminum reflector cup). Collecting spectra in the transflectance mode has some advantages over the transmission mode; for example, the ease of sample preparation and cleaning of the cup, as compared to the cuvette or glass vial, which needs to have a small path length. Another important advantage of transflectance cups is that they allow the sample to be rotated, enabling the greatest possible amount of sample to be scanned and the lack of sample homogeneity to be overcome [12].

In most of the scientific research papers published to date, samples were analyzed with the transmission mode and statically, not allowing the samples to be moved, using a range of presentation equipment (liquid cell holders, cuvettes and vials) [11,13,14,15,16,17]. One exception was a study by Manley and Eberle [18] comparing two different Fourier Transform Near-Infrared (FT-NIR) instruments and two analytical modes (transmittance, in static mode *versus* transflectance, in spinning mode). However, the authors offered no conclusions as to whether the static or spinning modes yielded more robust predictive models. 

The main aim of this study was therefore to systematically compare two basic modes of analysis (spinning *versus* static), using the same olive oil calibration and validation sets, for predicting acidity, peroxide index, K_232_, K_270_, alkyl and ethyl ester content, moisture and volatile matter content and insoluble impurities in light petroleum. 

## 2. Materials and Methods

### 2.1. Sampling

A total of 478 olive oil samples were used in this study. The samples, which included several olive varieties, were obtained from mills located all over Spain; they were received in sealed dark-green plastic 250-mL bottles and stored under refrigeration (5 °C, 85% RH), pending collection of NIR spectra the following day.

### 2.2. Reference Data 

The physico-chemical analysis of samples was performed in three accredited laboratories, highly specialized in olive oil analysis. Measurements of acidity, the peroxide index, K_232_, K_270_ and alkyl and ethyl ester content were carried out using the analytical methods described in [19]. Acidity and the peroxide index were determined by titration and results were expressed in terms of % of oleic acid and meq/kg, respectively. The extinction coefficients K_232_ and K_270_ were measured by UV spectrophotometric analysis at the specific wavelengths of 232 and 270 nm and expressed in terms of absorbance units (AU). The alkyl and ethyl esters were quantified by gas chromatography and expressed as mg/kg.

Moisture and volatile matter content were analyzed in accordance with [20]. Insoluble impurities in light petroleum were measured in accordance with [21].

### 2.3. Vis/NIR Spectra Acquisition

Spectra were collected for all samples in transflectance mode (log (1/R)) using two NIR instruments: (1) a FNS-6500 SY-I scanning monochromator (FOSS NIRSystems, Silver Spring, MD, USA); and (2) a FNS-6500 SY-II scanning monochromator (FOSS NIRSystems, Silver Spring, MD, USA). A folded-transmission gold reflector cup, diameter 3.75 cm, with a path length of 0.1 mm was used in both instruments. 

The FNS-6500 SY-I (FNS-I) and FNS-6500 SY-II (FNS-II) monochromators provide absorbance readings between 400 and 2500 nm, in 2 nm steps. The FNS-I instrument is equipped with a spinning module that rotates the cup (spinning mode), while the FNS-II has a transport module and the cup has no rotation (static mode). In both cases, two spectra were collected per sample and averaged for subsequent processing. NIR spectral data were collected using the WinISI II software package version 1.50 (Infrasoft International, Port Matilda, PA, USA) [22].

### 2.4. Calibration Development and External Validation

The WinISI II software package version 1.50 was used for the chemometric treatment of data needed for calibration and validation [22].

Prior to performing NIR calibrations, the CENTER algorithm included in the WinISI II software package was used to analyze the structure and spectral variability of the sample population. This algorithm performs an initial principal component analysis (PCA) and then calculates the distance of each sample (spectrum) from the center of the population in an n-dimensional space, using the Mahalanobis distance (GH); samples with a statistical value greater than 3 were considered outliers or anomalous spectra [12,23].

This algorithm was applied in the visible and near-infrared spectral region, from 400 to 2500 nm. Combined Standard Normal Variate (SNV) and Detrending (DT) methods were used for scatter correction [24] together with the first-derivative treatment “1,5,5,1”, where the first digit is the number of the derivative, the second is the gap over which the derivative is calculated, the third is the number of data points in a running average or smoothing and the fourth is the second smoothing [25].

The initial sample set comprised all samples analyzed (*n* = 478 samples), except in the case of moisture and volatile matter content and insoluble impurities in light petroleum (*n* = 368 samples). Having ordered the sample set by spectral distances (from smallest to greatest distance from the center), those displaying GH values >3 were discarded as outliers. After discarding outliers, each initial sample set comprised 459 samples, except for moisture and volatile matter content and for insoluble impurities in light petroleum (*n* = 349 samples), since 19 samples were identified as outliers using both analysis modes. The structured sample set was subsequently used as the basis for establishing the calibration and validation sets to be used in constructing predictive models. One out of every four samples in the initial set was selected for the validation set, the remainder forming the calibration set. The samples comprising the calibration and validation sets were the same for the two instruments, in order to facilitate subsequent comparison of results.

NIR calibration models were then constructed to predict acidity, peroxide index, K_232_, K_270_, alkyl and ethyl ester content, moisture and volatile matter and insoluble impurities in light petroleum content, using Modified Partial Least Squares (MPLS) as the linear regression strategy [12]. All regression equations were obtained using SNV-DT for scatter correction [24]. Four derivate mathematical treatments were tested in the development of NIR calibrations, in the spectral region 400–2500 nm: 1,5,5,1; 2,5,5,1; 1,10,5,1 and 2,10,5,1 [25].

Six cross-validation groups were included in the regression process in order to select the optimum number of factors and avoid overfitting. Finally, validation errors were combined to obtain a standard error of cross validation (SECV) [26].

The statistics employed to select the best equations using MPLS were: the coefficient of determination for calibration (*r*^2^_c_), the standard error of calibration (SEC), the coefficient of determination for cross validation (*r*^2^_cv_) and the standard error of cross validation (SECV). However, other statistics such as the Residual Predictive Deviation (RPD), calculated as the ratio of the standard deviation (SD) of the reference data to the SECV and the Range Error Ratio (RER), defined by the ratio between the range of the reference data to the SECV, were also calculated. These statistics enable SECV to be standardized or converted to comparable units, facilitating the comparison of results obtained by other authors, using sets with different means, standard deviations and ranges from those used in the present paper [27]. 

Once the best predictive model for each parameter, analyzed using both forms of presentation and without the elimination of samples during the development of the models, had been selected by statistical criteria—and prior to external validation—tests were run for significant differences between models for each parameter, with a view to identifying the most appropriate sample presentation mode for routine use in olive oil laboratories. SECV values for the best equations obtained for each parameter with both instruments and both presentation modes were compared using Fisher’s F test [28,29]. Values for F were calculated as:

(1) $$F=\frac{{\left(SECV_{2}\right)}^{2}}{{\left(SECV_{1}\right)}^{2}}$$

where SECV_1_ and SECV_2_ are the standard error of cross validation of two different models and SECV_1_ < SECV_2_. F is compared to F_critical (1 − *P*, n − 1, n − 1)_ read from the table with *P* = 0.05 and n − 1 degrees of freedom. If F is higher than F_critical_, the two SECV values are significantly different. 

Once the best sample presentation method was selected and given the importance of the construction of robust predictive models [27,30], Student’s T test was used to detect and eliminate those samples considered physico-chemically anomalous. The best predictive models obtained were subsequently subjected to external validation according to the protocol outlined [31,32] based on the following statistics: standard error of prediction (SEP), standard error of prediction corrected for bias (SEP(c)), bias and coefficient of determination for external validation *(r*^2^_v_). This statistical process is based on the determination of a known significant error, termed ‘bias’ and an unexplained significant error, termed standard error of prediction bias-corrected (SEP(c)). Generally, for calibration groups comprising 100 or more samples and validation groups containing nine or more samples, the following control limits are assumed: Limit Control SEP(c) = 1.30 × SEC, Limit Control bias = ±0.60 × SEC, minimum value of 0.6 for *r*^2^_v_ and slope value between 0.90–1.1.

## 3. Results and Discussion

### 3.1. Spectral Features

**Figure 1:**
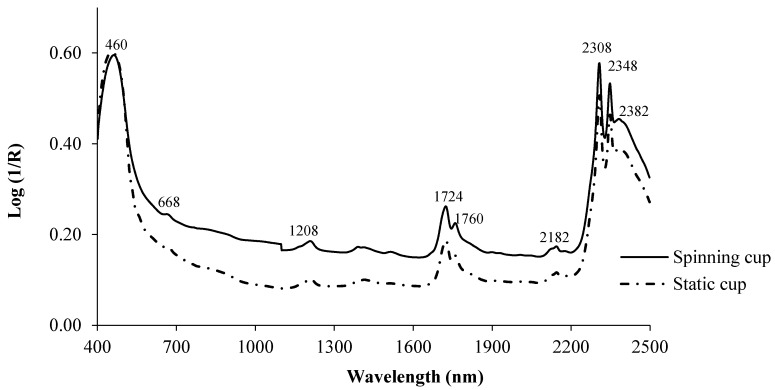
Log (1/R) for olive oil using the spinning and static cups.

Typical log (1/R) spectra, together with the most relevant absorption bands for olive oil samples scanned in the static and spinning cups are shown in Figure 1.

Both scanning modes produce spectra with similar shapes. In the visible region, the highest absorbance was recorded at wavelengths of between 420–460 nm and also at 668 nm, due to the presence of pigments, mainly carotenoids and anthocyanins and chlorophyll, which are responsible for yellow and green coloring, respectively [33]. In plant-tissue studies it has been reported that chlorophyll has a single and fairly sharp absorption band at 675 nm [33]. Garrido-Varo et al. [9] for a set of 105 olive oils varying within a range of yellow-green hues, also reported relevant absorption peaks in the 424–462 nm and 668–670 nm regions. 

In the NIR region (Figure 1), the highest absorption peaks were detected at 1208, 1724, 1760, 2182, 2308, 2348 and 2382 nm. Early band assignment studies using a variety of vegetable oils concluded that the bands at around 1208 nm are attributable to the second overtone, relating to the stretching of C-H molecular bonds, while those at 1724 and 1760 nm are assignable to the first overtone of C-H bond vibrations in the methyl, methylene and ethylene groups [7].

Garrido et al. [7] reported that absorbance at 2182 nm could be due to N-H band absorption, whilst peaks at 2308, 2348 and 2382 nm derive from combination bands stemming from the stretching of the C-H molecular bonds of fatty acids. Chen and Chen [34] reported that the wavelengths showing the strongest correlation with the color of various vegetable oils were 850, 870 and 1310 nm for yellow and 400, 521 and 860 nm for red.

### 3.2. Descriptive Data for NIR Calibrations and Validations

Values for the number of samples, range, mean, standard deviation (SD) and coefficient of variation (CV) for each of the parameters analyzed for the calibration and validation sets after the application of the CENTER algorithm and the removal of spectral outliers are shown in Table 1.

It should be stressed that structured selection, using only spectral information treatment algorithms such as CENTER, proved adequate and useful, since the calibration and validation sets displayed similar values for mean, range and standard deviation for all study parameters and the ranges for the validation set lay within the ranges recorded for the calibration set.

It is also worth noting that the parameters displaying the greatest variability were acidity, alkyl and ethyl ester content and insoluble impurities in light petroleum, all of which recorded a coefficient of variation of over 90%. By contrast, the least variable parameters were K_232_ and K_270_, with values below 25%. The great variability observed in the physico-chemical parameters could be attributable to the fact that the oils belonged to different categories (EVOO, VOO and LOO), from olives grown in different geographical areas, harvested at different times, extracted by different methods (two- and three-phase extraction systems), gathered both from the ground and from the tree, at various stages of ripening and in varying states of health.

### 3.3. Comparison of Sample Presentations

As Table 2 shows, significant differences (*p <* 0.05) were detected between the dynamic method of sample presentation (spinning cup) and the static presentation for the prediction of acidity, K_232_ and insoluble impurities in light petroleum. 

Most NIR laboratories dedicated to food analysis and particularly those specializing in oils, tend to purchase a NIR instrument with a given sample presentation mode for constructing models to predict quality parameters. The results obtained here suggest that dynamic presentation using a spinning cup module is the most suitable mode for this purpose.

### 3.4. Calibration for Predicting Physico-Chemical Quality Parameters in Olive Oil

After comparison of sample presentation modes using the same number of samples, new calibration models were constructed, but this time removing physico-chemical outlier samples; only the FNS-6500 SY-I with spinning module and rotating cup was used for this purpose. Calibration statistics for the best models for the prediction of physico-chemical parameters in olive oil are shown in Table 3.

The models displaying the greatest predictive capacity were constructed using the first derivative of the spectrum, except for the parameters of acidity and insoluble impurities in light petroleum, where the second derivative provided better results.

Shenk and Westerhaus [32] note that the goodness of fit of calibrations for which *r*^2^_cv_ ≥ 0.90 and *r*^2^_cv_ = 0.70–0.89 may be regarded as excellent and good, respectively, whereas values of *r*^2^_cv_ between 0.50 and 0.69 enable classification between high, medium and low values of the parameters being analyzed. In accordance with this, depending on the values obtained for *r*^2^_cv_, the equations derived for acidity, peroxide index, alkyl and ethyl esters may be considered as having excellent (acidity) and good predictive capacity (the rest of aforementioned parameters) and the equations for K_232_, K_270_, moisture and volatile matter content and insoluble impurities in light petroleum may be considered as sufficient for carrying out a classification between high, medium and low values of the said parameters.

As Fearn [35] points out, while the *r*^2^_cv_ statistic can be a useful measure of the performance of a calibration, it does have limitations. One major constraint is its dependence on the range of values—and on the standard deviation (SD) of the reference values—of the calibration set. This would account for the lower *r*^2^_cv_ values recorded here for K_270_, moisture, volatile matter content and insoluble impurities in light petroleum compared to the *r*^2^_cv_ values obtained for the other parameters being studied. 

Fearn [35] and Dardenne [36] have also shown that the RPD statistic used in most NIR research is equal to 1/√(1 − *r*^2^_cv_) and depends to the same degree as *r*^2^_cv_ on the range of the data in the calibration. This view is borne out by the results obtained here (Table 3), which indicate a close match between the highest and lowest r^2^_cv_ values and RPD for some parameters. Thus, whereas for the acidity parameter, which exhibits a high *r*^2^_cv_ value (0.98), the RPD is 7.70, for the other parameters, with *r*^2^_cv_ values ranging from 0.53–0.62, the RPD ranges from 1.46–2.24. 

The RPD statistic is widely used in NIR research for assessing the efficiency of NIR predictions. RPD values between 3 and 5 indicate efficient NIR predictions [27], although such values were established when the analysis with NIR instruments was carried out on dry and finely-ground products, using laboratory instruments. In current working conditions, however, with liquid products that are analyzed intact, lower values of RPD would often be acceptable for an equation to be considered suitable for routine use. Furthermore, Esbensen et al. [37] have recently argued that RPD values depend on the kind of sample, on its prior preparation, on the way it is presented to the instrument, on the error of the reference method and, in general, on the variance within the sample set used for calibration. Notwithstanding this, RPD is a highly useful statistic in that it enables the comparison of predictive models produced by distinct groups, which in consequence have different means and standard deviations.

Having noted this and considering the constraints regarding *r*^2^_cv_ and additionally bearing in mind that the values of SEC and SECV cannot be compared in absolute terms, given that they are dependent on the mean value of the parameter to be predicted, a discussion of the results relative to those obtained by other authors follows, attempting to standardize the values of SEC and SECV, with such statistics as RPD and RER.

Results similar to those obtained here for acidity were reported by Garrido-Varo et al. [9] using a monochromator allowing sample movement during NIR analysis and also by [13,16,38] with static sample presentation using monochromators and FT-NIR instruments. It should be noted, however, that the latter two authors recommend heating the sample prior to NIR analysis. Nevertheless, previous research by the present authors [9,39] has shown that the main sources of variation in the results of NIR analysis of virgin olive oil and animal fat are associated not with the temperature but with the path length of the cuvette or cup and the availability of a repeatability file. NIR equations with strong predictive capacity can be obtained without heating the sample, thus saving time and effort during analysis, as well as ensuring that sample physico-chemical and sensory characteristics remain unimpaired. Cayuela-Sánchez et al. [14] and Inarejos-García et al. [16], using diode-array and FT-NIR instruments respectively with static sample presentation, reported RPD values of 3.14 and 1.54, respectively, both considerably lower than those obtained here. 

The findings emerging from the present study with regard to acidity are of particular interest, since the prediction of this parameter is a crucial aspect of measuring the quality of virgin olive oil. Biologically-synthesized fat is neutral, i.e., the oil in a healthy olive on the tree contains 0% acidity. 

Although current EU legislation [4] establishes a maximum acidity limit of 0.8% for extra virgin olive oil, in practice acidity levels in bottled oils tend to be well below this figure, generally lying between 0.2% and 0.5%. A very low acidity index is consistent with high quality; values close to 0.1% indicate the use of properly-handled olives in a perfect state [5].

For the peroxide index, the results were better than those reported by Garrido-Varo et al. [9] (RPD = 1.72), but slightly poorer than those obtained by Manley and Eberle [18] (RPD = 2.77 with spinning mode presentation and RPD = 3.52 with static presentation), Armenta et al. [38] (RPD = 3.3), Cayuela-Sánchez et al. [15] (RPD = 2.84) and Inarejos-García et al. [17] (RDP = 2.63). Gertz [16], using a group of 308 samples, obtained an equation of considerable accuracy (*r*^2^_cv_ = 0.94, RER = 24.49), compared to that obtained in the present study, probably attributable to a lesser degree of laboratory error, given that the samples used by this author were analyzed in a single laboratory, whereas the samples in the present study, owing to the cases involved, were analyzed in three distinct accredited laboratories and it is known that the use of data from different laboratories may reduce the precision and exactitude of the models [40]. 

The peroxide index measures the presence of peroxides in olive oil. These are the first products of fatty acid oxidation and are used as indicators of oil quality and stability, since oxidation is one of the primary causes of quality loss in olive oil [1,38,41]. The peroxide index indicates primary oxidation status before a rancid smell or taste become apparent and also potential deterioration of certain components of nutritional interest, such as vitamin E [5].

A detailed discussion of the difficulties encountered by other authors in making an accurate prediction of this analytical parameter in a range of raw, oxidized and fried vegetable (palm, sesame, peanut, sunflower) oils may be found in [7]. According to the aforementioned authors, one reason underlying the poor predictive capacity obtained by some authors is the inherently low repeatability and reproducibility of the peroxide value reference method [42,43]. Another reason may be attributed to the fact that the peroxide index reference method provides a measure of the current rancidity of a fat or oil and this state may vary with storage and/or handling of the sample until NIR analysis is performed. It is therefore advisable that the reference analysis and the NIR spectral analysis are carried out at the same time. 

Like the peroxide value, the K_232_ index is a useful means of highlighting initial oxidation, by quantifying light absorption in the ultraviolet (UV) region at wavelength 232 nm. Upper limits of ≤ 2.50 and ≤2.60 have been set for extra virgin and virgin olive oil respectively [4]. The K_270_ index detects a more advanced state of oxidation. As the oxidative process advances, peroxides are modified to give α-diketones or α-unsaturated ketones, which absorb UV light at a different wavelength (270 nm) from hydroperoxides [5,44].

Thus, for reasons similar to those already mentioned for the peroxide index, the equations obtained for other parameters indicative of oxidation state, such as K_232_ and K_270_, account for 62% and 56% respectively of the variation detected in these analytical parameters.

The results shown in Table 3 for K_232_ and K_270_ are better than those reported by Inarejos-García et al. [17] (*r*^2^_cv_ of 0.40 and 0.54; RPD = 1.09 and 1.19 for K_232_ and K_270_, respectively). However, they are slightly poorer than those recorded by Garrido-Varo et al. [9] for K_232_ (RPD = 1.71) and greater in the case of K_270_ (RPD = 2.00). 

Gertz [16] using a group of virgin olive oil samples with characteristics more akin to those used in the present study, albeit with a smaller number of samples (K_232_ = 133 samples; K_270_ = 101 samples), obtained equations accounting for 92.1% and 92.4% of the variance, respectively, with a standard error of calibration (SEC) of 0.088 AU and 0.009 AU for the aforementioned parameters, respectively, very similar to those obtained in the present study (Table 3). The predictive capacity of the models designed to predict alkyl esters (*r*^2^_cv_ = 0.74; SECV = 19.52 mg/kg) and ethyl ester content (*r*^2^_cv_ = 0.73; SECV = 12.75 mg/kg) may be rated as good, in accordance with [26]. 

No references have been found in the literature to the prediction of alkyl and ethyl esters in virgin olive oil using NIR technology. However, measurement of these esters can serve to distinguish olive oil from olive-pomace oil, as well as to confirm extra virgin olive oil quality by detecting fraudulent mixtures of extra virgin olive oil with lower quality oils [6]. In April 2011, EU Regulation n. 61/2011 [45] came into force and Fatty Acid Alkyl Ester (FAAE) content became an official quality parameter for extra virgin olive oil. Methyl and ethyl alcohols, which constitute part of these esters, are formed as a result of enzymatic and fermentative processes that take place if overripe or incorrectly-stored olives suffer damage to their cellular structures prior to crushing. If this occurs, the leakage of water can pave the way to processes capable of generating a wide range of volatile substances, including ethanol. It is no coincidence that “viny-vinegary” observations, linked to alcoholic, lactic and acetic fermentations, are one of the defects that downgrade extra-virgin olive to virgin or lampante olive oil, depending on their intensity. 

The EU Regulation [4] established limits for FAEES of 40, 35 and 30 mg/kg for 2013–2014, 2014–2016 and crop years post-2016, respectively.

Over recent years, growing consumer appreciation of the nutritional and organoleptic qualities of virgin olive oil and insistence that these should be preserved until the oil is consumed, has prompted a search for new criteria for studying and defining extra virgin olive oil quality and interest has focused on moisture and impurity levels. The non-destructive measurement of moisture and volatile matter levels is of particular interest to the olive oil industry, because the presence of moisture facilitates oxidative rancidification, giving rise to off-flavors and off-smells [5].

Models constructed for moisture and volatile matter content and for insoluble impurities in light petroleum yielded *r*^2^_cv_ values of 0.53 and 0.61 and SECV values of 0.03 and 0.02% m/m, respectively. It should be noted that fewer samples were used in the construction of models for both these quality parameters.

As far as the present authors are aware, only one published study makes reference to the prediction of moisture and volatile content using NIR [9]. If the aforementioned authors’ results are compared with those shown in Table 3, it becomes apparent that the lower *r*^2^_cv_ value (0.53) obtained in the present study may be owing to the lower range and SD of the calibration set, mainly consisting of extra virgin and virgin oils. The SECV values are in both cases very low and therefore confirm that the NIR analysis of this important analytical parameter may be carried out with a high degree of certainty.

No references have been found to the measurement of insoluble impurities in light petroleum using NIR technology, another parameter of importance for the olive oil extraction industry, since impurities—in the form of mineral substances, nitrogenized substances, resins and oxidized fatty acids—may precipitate and ferment, giving rise to the characteristic “amurca” or “olive-lees” flavor, deriving from the bitter-tasting, dark-colored, watery sediment that settles out of unfiltered olive oil over time [5].

### 3.5. Validation for Predicting Physico-Chemical Quality Parameters in Olive Oil

**Figure 2:**
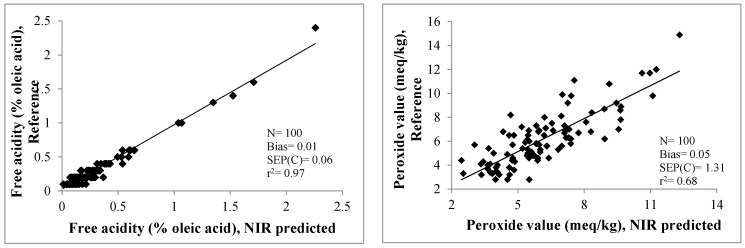

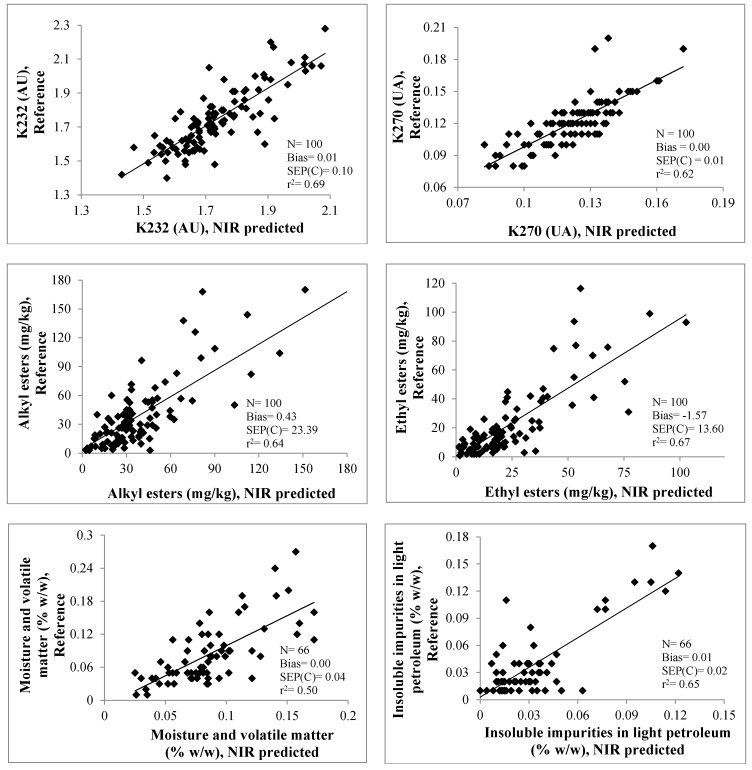
Reference versus NIR-predicted data for the validation set. Instrument FNS-6500 SY-I. Sample presentation in spinning mode.

Validation statistics for the prediction of quality parameters in olive oils using the FNS-6500 SY-I instrument with spinning sample presentation, together with the control limits recommended by [31,32] are shown in Table 4. In addition, the representation of the reference values *versus* the NIR predicted values of each parameter is shown in Figure 2. 

SEP is recognized as the best statistic for evaluating the predictive capacity of an equation since, as Fearn [46] points out, SEP is a direct measurement of how well the calibration is likely to perform in future samples. A widely-accepted criterion for useful NIR equations is that SEP should be less than two times Standard Error of Laboratory (SEL) [31]. 

Unfortunately, the authors of the present study were unable to estimate the value of SEL and analyses of a single analytical parameter were moreover conducted at different laboratories. Therefore, given the lack of their own information about SEL, reference values for this statistic obtained from other authors using mean data were employed as follows: acidity (SEL = 0.10% of oleic acid [47]); peroxide index (SEL =1.41 meq/kg [17]); k_232_ (0.42 AU [17]); k_270_ (0.048 AU [17]).

Regardless of differences in analysis mode, the instrument used, the spectral region or characteristics of the calibration and validation sets employed, bearing in mind that the error of the reference method is implicit in the SEP, the differences found between the data obtained in the present study and the other authors should be attributed to differences in the SEL [48]. In addition, the present study uses not only the standard error of laboratory (SEL) statistic, but also the Standard Error of Difference (SED) between reference values obtained from different laboratories [32]. It is therefore possible that this is one of the reasons accounting for the fact that Gertz [16] obtained better results than those of the present study, given that he conducted the reference analysis in a single laboratory, whereas in the present study three accredited laboratories were responsible for carrying out the reference analysis. Although these laboratories specialize in the analysis of olive oil, error deriving from the differences between them, which in analytical terms is known as the reproducibility and repeatability of the method, should be borne in mind.

Meanwhile, as is evident from Table 4, the models constructed for all parameters, except for moisture and volatile matter, met validation requirements in terms of *r*^2^_v_ (*r*^2^_v_ > 0.6) and both SEP(c) and bias values were within confidence limits: the equations thus ensure accurate prediction and can be applied routinely. The comparatively low *r*^2^_v_ value displayed for moisture and volatile matter may be due to the narrower range and lower SD recorded for this parameter (Table 1). This is also clearly illustrated in Figure 2 where it is evident that moisture and volatile matter exhibited by most samples lie in the range 0.01–0.1% m/m, with very little coverage of the range for other values.

However, although the value of *r*^2^_v_ does not meet the limit recommended for routine application (*r*^2^_v_ > 0.60) for the moisture and volatile matter parameters, it should be noted that SEP(c) was close to confidence limits and bias and slope were below these limits, suggesting that the NIR equations constructed here may be regarded as a first step in the fine-tuning of NIR technology for the monitoring of chemical parameters in the olive oil industry. 

It must be stressed that for the extreme high values of the parameters acidity and insoluble impurities in light petroleum shown in the Figure 2 correspond to LOO category.

## 4. Conclusions

The models developed here for predicting and classifying a number of olive oil quality parameters highlight the potential of NIR technology as a non-destructive tool for quality control during production and storage in the olive oil industry, requiring no previous sample preparation; slight differences in model robustness were recorded between spinning and static sample presentation. 

Finally, in the authors’ opinion, the organizations responsible for the inspection and classification of oils in producer countries and the International Olive Council itself, should take into consideration that, while the number of scientific publications such as the present one is considerably lower than those carried out involving other analytical methods, the results obtained in this and the other studies referred to are sufficiently robust to support collaborative studies at a worldwide level; these would show that it is possible to obtain robust models for a considerable number of physico-chemical parameters specified as quality criteria in the current regulatory framework. This in turn would contribute to the real possibility of increasing the number of production samples inspected every year and a highly significant diminution of the analytical cost and time needed to carry out such inspections. 

## Figures and Tables

**Table 1:** Statistical analysis of calibration and validation sample sets: data range, mean, standard deviation (SD) and coefficient of variation (CV).

Parameter	Set	Number of Samples	Range	Mean	SD	CV (%)
Free acidity (% in oleic acid)	Calibration	359	0.10–5.70	0.41	0.64	156.10
	Validation	100	0.10–2.40	0.33	0.33	100.00
Peroxide value (meq/kg)	Calibration	359	1.60–44.50	6.30	3.82	60.63
	Validation	100	2.80–14.90	6.14	2.29	37.30
K_232_ (AU)	Calibration	359	1.37–5.42	1.76	0.31	17.61
	Validation	100	1.40–2.28	1.74	0.18	10.34
K_270_ (AU)	Calibration	359	0.07–0.41	0.13	0.03	23.08
	Validation	100	0.08–0.20	0.12	0.02	16.67
Alkyl esters (mg/kg)	Calibration	359	3.00–610.00	52.07	72.76	139.73
	Validation	100	3.00–170.00	39.67	36.80	92.77
Ethyl esters (mg/kg)	Calibration	359	1.00–461.00	29.24	49.20	168.26
	Validation	100	1.00–116.40	21.36	23.57	110.35
Moisture and volatile matter (% m/m)	Calibration	283	0.01–0.63	0.09	0.06	66.67
	Validation	66	0.01–0.27	0.08	0.06	75.00
Insoluble impurities in light petroleum (% m/m)	Calibration	283	0.01–0.31	0.04	0.05	125.00
	Validation	66	0.01–0.17	0.04	0.04	100.00

**Table 2:** Comparison between SECV values obtained for the best models for predicting olive oil quality parameters using the FNS-6500 SY-I monochromator with spinning sample presentation and the FNS-6500 SY-II monochromator with static sample presentation; Fisher test (*p* ≤ 0.05).

Parameter	SECV Spinning Cup	SECV Static Cup	F	F_critical_
Acidity (% in oleic acid)	0.074	0.070	1.14	1.09
Peroxide index (meq/kg)	2.675	2.615	1.05	1.09
K_232_ (AU)	0.196	0.208	1.13	1.09
K_270_ (AU)	0.022	0.022	1.02	1.09
Alkyl esters (mg/kg)	46.71	44.79	1.08	1.09
Ethyl esters (mg/kg)	34.69	33.53	1.07	1.09
Moisture and volatile matter (% m/m)	0.053	0.052	1.04	1.10
Insoluble impurities in light petroleum (% m/m)	0.037	0.040	1.13	1.10

**Table 3:** Calibration statistics for the best models for predicting quality parameters in olive oil. FNS-6500 SY-I monochromator with dynamic (spinning module) sample presentation

Parameter	N	PLS Terms	Mean	SD	SEC	*r*^2^_c_	SECV	*r*^2^_cv_	RPD	RER
Acidity (% oleic acid) ^1^	348	16	0.36	0.47	0.05	0.99	0.06	0.98	7.70	38.33
Peroxide index (meq/kg) ^2^	345	12	5.97	2.82	1.17	0.83	1.40	0.76	2.02	8.64
K_232_ (AU) ^3^	342	11	1.72	0.18	0.09	0.75	0.12	0.62	1.50	7.33
K_270_ (AU) ^4^	344	11	0.12	0.02	0.01	0.67	0.01	0.56	2.24	12.00
Alkyl esters (mg/kg) ^5^	334	9	38.97	37.80	17.36	0.79	19.52	0.74	1.94	8.56
Ethyl esters (mg/kg) ^6^	340	12	21.58	24.43	10.91	0.80	12.75	0.73	1.92	9.05
Moisture and volatile matter (% m/m) ^7^	267	11	0.08	0.05	0.02	0.71	0.03	0.53	1.50	8.67
Insoluble impurities in light petroleum (% m/m) ^8^	260	6	0.03	0.03	0.02	0.71	0.02	0.61	1.46	8.00

Math Treatment: ^1^: SNV + DT—2,10,5,1; ^2^: SNV + DT—1,10,5,1; ^3^: SNV + DT—1,5,5,1; ^4^: SNV + DT—1,10,5,1; ^5^: SNV + DT—1,10,5,1; ^6^: SNV + DT—1,10,5,1; ^7^: SNV + DT—1,10,5,1; ^8^: SNV + DT—2,5,5,1.

**Table 4:** Validation statistics for predicting quality parameters in olive oil using the FNS-6500 SY-I monochromator with dynamic (spinning module) sample presentation.

Parameter	N	SEP	Bias	Bias Limit	SEP_(C)_	SEP_(C)_ Limit	*r*^2^_v_	Slope
Acidity (% oleic acid)	100	0.06	0.01	±0.03	0.06	0.07	0.97	0.95
Peroxide index (meq/kg)	100	1.31	0.05	±0.70	1.31	1.52	0.68	0.92
K_232_ (AU)	100	0.10	0.01	±0.05	0.10	0.12	0.69	1.10
K_270_ (AU)	100	0.01	0.00	±0.01	0.01	0.01	0.62	1.04
Alkyl esters (mg/kg)	100	22.29	0.43	±10.42	22.39	22.57	0.64	0.91
Ethyl esters (mg/kg)	100	13.63	−1.57	±6.55	13.60	14.18	0.67	0.96
Moisture and volatile matter (% m/m)	66	0.04	0.00	±0.01	0.04 *	0.03	0.50 *	1.09
Insoluble impurities in light petroleum (% m/m)	66	0.02	0.01	±0.01	0.02	0.03	0.65	1.09

* Values exceeding control limits.
